# Exploring the Role of Intrinsic Motivation in Healthy Eating Intentions: An Extension of the Theory of Planned Behavior in Chinese Adults

**DOI:** 10.3390/nu17122007

**Published:** 2025-06-15

**Authors:** Xiaoyu Ma, Seungwoo Lee, Ji-Yun Hwang

**Affiliations:** 1 Department of Foodservice Management and Nutrition, Graduate School, Sangmyung University, Seoul 03016, Republic of Korea; maxiaoyu@taru.team; 2 Major of Foodservice Management and Nutrition, Sangmyung University, Seoul 03016, Republic of Korea; slee@smu.ac.kr

**Keywords:** healthy eating, motivation, Chinese adults, behavior

## Abstract

**Background:** Unhealthy diets are a leading cause of obesity, which increases the risk of chronic diseases such as hypertension, type 2 diabetes, and cardiovascular conditions. The theory of planned behavior (TPB) explains eating intentions through attitudes, subjective norms, and perceived behavioral control (PBC), yet these constructs may not fully account for the complexity of intention formation. Motivation has been identified as a stronger predictor of the maintenance of long-term healthy behaviors. This study extends the TPB by introducing motivation as a mediating variable to examine whether attitudes and subjective norms influence motivation, which in turn affects behavioral intention. **Methods:** An online survey was conducted between 2019 and 2023, collecting responses from 2114 adults residing in Beijing, Shanghai, and selected regions of Anhui, Daqing, and Henan. Structural equation modeling (SEM) was employed to examine the relationships among the TPB constructs, motivation, and behavioral intention. **Results:** SEM analysis revealed significant associations between attitude and subjective norms with motivation. Additionally, motivation and PBC were significantly associated with behavioral intention. Motivation was found to mediate the relationships between attitude and intention (95% confidence interval (CI): 0.004–0.021, *p* = 0.004) and subjective norms and intention (95% CI: 0.013–0.035, *p* = 0.012). **Conclusions:** These findings suggest that attitudes and subjective norms enhance motivation for healthy eating among Chinese adults. In turn, motivation—along with PBC—plays a key role in predicting behavioral intention. Future research should further explore the mediating role of motivation in shaping healthy eating intentions within the TPB framework.

## 1. Introduction

Global trade liberalization, economic growth, and rapid urbanization have profoundly transformed people’s living environments, dietary habits, and lifestyles [1]. The increasing consumption of processed and convenience foods, coupled with the rise in dining out—particularly at fast-food establishments—has become a hallmark of modern life [2,3]. However, eating outside the home often limits individuals’ ability to control or understand the nutritional content and quality of their meals [4]. Compared to home-cooked foods, meals consumed away from home are more likely to be high in calories and fat, contributing to unhealthy dietary patterns that pose a significant global public health challenge [5,6,7].

In China, the world’s most populous country, obesity has become a pressing concern. According to a 2023 national survey conducted by the PLA General Hospital, 34.8% of Chinese adults were classified as overweight and 14.1% as obese [8]. The health implications of overweight and obesity are wide-ranging, increasing the risk of chronic conditions such as hypertension, type 2 diabetes, and cardiovascular disease [5,6,7]. Additionally, individuals living with obesity often face weight-based stigma and discrimination, which can adversely affect both mental and physical health [9]. This stigma may lead to physiological imbalances, including elevated cortisol and inflammatory markers [10,11], as well as symptoms of depression and anxiety [12,13].

Given the complexity of obesity and its multifaceted consequences, there is an urgent need for comprehensive and multi-level public health strategies. While many interventions have focused on improving the food environment, enhancing access to nutritious options, and promoting physical activity [14], emerging evidence highlights the critical role of behavioral motivation—particularly intrinsic motivation—in maintaining healthy behaviors over time [15,16].

Intrinsic motivation refers to engaging in behaviors for inherent enjoyment, interest, or personal satisfaction. Recent research further suggests that it may also arise from perceiving health-related behaviors as meaningful steps toward personal goals, thereby enhancing persistence and psychological commitment [17]. In the context of healthy eating, intrinsic motivation may be shaped by experiences such as enjoyment of food, autonomy in food choices, and a sense of accomplishment in maintaining a nutritious diet.

Cultivating such motivation is increasingly recognized as key to effective and sustainable obesity prevention strategies [18,19]. Without a deeper understanding of consumers’ motivational drivers, public health interventions may fall short of producing meaningful behavioral change [20]. Notably, recent national efforts in China have begun to incorporate motivation-focused approaches. The China Adult Obesity and Nutrition Guidelines (2024) [21], for instance, promote culturally sensitive meal plans, such as low-fat adaptations of Sichuan cuisine or warming northern dishes, to preserve culinary satisfaction while improving diet quality [21]. In addition, the 2024 launch of the Resident Electronic Health Records system by the National Health Commission enables individuals to monitor real-time changes in health metrics, thereby reinforcing self-efficacy and a sense of progress in lifestyle changes [22].

Despite these advances, few studies have empirically examined how intrinsic motivation interacts with established psychological constructs to shape healthy eating intentions among Chinese adults. The theory of planned behavior (TPB) has been widely used to predict health-related behaviors through three key constructs: attitudes, subjective norms, and perceived behavioral control (PBC). However, individuals may hold positive attitudes toward a behavior or experience social pressure to engage in it without necessarily acting unless they are also motivated by personal relevance or expected outcomes [23]. Therefore, integrating motivation into the TPB framework may provide a more comprehensive understanding of how healthy eating intentions are formed.

While most previous research has focused on specific cities in northern or southern China, large-scale national data reveal regional disparities in the prevalence of overweight and obesity—generally higher in northern regions and among men [8]. This study addresses these gaps by incorporating regional differences and exploring the role of intrinsic motivation as a mediator within an extended TPB model. This study aims to: first, validate a measurement model using confirmatory factor analysis (CFA) to ensure the structural reliability and internal consistency of TPB constructs and motivation among Chinese adult; second, extend the TPB framework by incorporating intrinsic motivation and examining its mediating role in the relationships between attitudes, subjective norms, and behavioral intention; and, last, develop and test an integrated structural equation model (SEM) to explore how motivation contributes to healthy eating intentions and to inform the design of more effective, motivation-oriented public health interventions.

## 2. Materials and Methods

### 2.1. Settings and Participants

The study protocol is detailed in our previous research [24,25,26]. The survey targeted adults residing in five cities in China: Beijing, Shanghai, certain regions of Anhui Province, Henan Province, and Daqing. Data collection occurred from 2019 to 2023, with five surveys conducted across these cities. A snowball sampling method was used to distribute the questionnaire via QR codes or through links on the WeChat platform. A total of 2232 Chinese adults participated in the online survey, which included incentive information and offered rewards as compensation. To ensure questionnaire reliability, 118 respondents were excluded based on five criteria. Ultimately, 2114 eligible respondents comprised the final dataset. This study received institutional review board (IRB) approval from Sangmyung University for the years 2019 through 2023 (IRB approval numbers: BE2019-01-05, SMUIRBC-2020-009, SMUIRBC-2023-001).

### 2.2. Conceptual Framework

**Figure 1:**
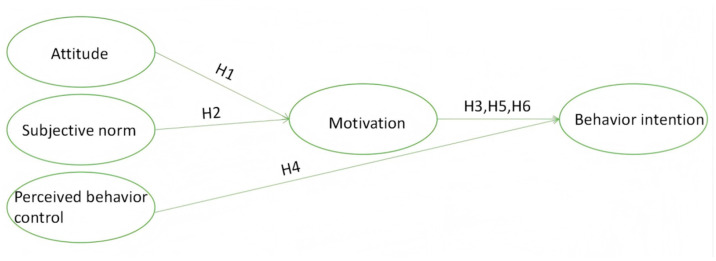
Theory of planned behavior.

To elucidate the relationships among the three core components of the TPB—namely, attitude, subjective norms, and PBC—as well as motivation and healthy dietary behavior intentions, this study employed Ajzen’s conceptual framework (Figure 1) [23]. The following hypotheses were proposed based on this framework: first, attitude and subjective norms significantly influence motivation; second, PBC and motivation significantly affect healthy dietary intentions; and, third, motivation mediates the relationship between attitude, subjective norms, and behavioral intentions.

**Hypothesis** **1** **(H1).** *Attitudes have a significant effect on motivation.*

**Hypothesis** **2** **(H2).** *Subjective norms have a significant effect on motivation.*

**Hypothesis** **3** **(H3).** *Motivation has a significant effect on healthy eating intentions.*

**Hypothesis** **4** **(H4).** *Perceived behavioral control has a significant effect on healthy eating intentions.*

**Hypothesis** **5** **(H5).** *Motivation mediates the relationship between attitudes and healthy eating intentions.*

**Hypothesis** **6** **(H6).** *Motivation mediates the relationship between subjective norms and healthy eating intentions.*

### 2.3. Survey Instrument

The development process of the research questionnaire has been extensively detailed in our previous studies [24,25,26]. The questionnaire was subsequently translated into Chinese by experts, based on the context of Chinese adults, and its final version was confirmed through preliminary testing with Chinese students majoring in nutrition. Notably, to ensure the validity and applicability of the questionnaire, we employed instruments that had already been validated in adolescent populations [27,28]. We observed high explanatory power of the research questionnaire among Chinese adults [24,25,26], which provided a robust foundation for subsequent data collection and analysis.

The questionnaire included demographic data (age and gender). Body mass index (BMI) was calculated by dividing an individual’s weight (in kilograms) by the square of their height (in meters) (kg/m^2^), with height and weight data derived from self-reported measurements. For each participant, BMI classifications were established according to the criteria developed by the World Health Organization (WHO) Regional Office for the Western Pacific (WPRO), specifically for Asian adult populations. The weight status categories were defined using the following BMI thresholds: underweight (<18.5 kg/m^2^), normal weight (18.5–22.9 kg/m^2^), overweight (23.0–24.9 kg/m^2^), and obese (25.0–29.9 kg/m^2^). These anthropometric measurements were normalized based on age and sex distributions to ensure appropriate comparative analysis across demographic groups [29,30].

In our previous research, the key components of the TPB—namely, attitude, subjective norms, PBC, and motivation, as well as healthy dietary behavior intentions—were well described. Internal consistency was verified using Cronbach’s alpha coefficients: attitude was measured with six items (α = 0.950), subjective norms with eight items (α = 0.950), PBC with four items (α = 0.905), and healthy dietary intentions with four items (α = 0.939) [24,25,26].

Motivation was assessed through six items [31,32,33]. Respondents rated their agreement on a 10-point Likert scale (1 = Strongly Disagree to 10 = Strongly Agree) for statements related to motivation. The six questions were as follows: “How important is maintaining a healthy diet to you?”, “How confident are you in your ability to maintain a healthy diet?”, “How ready are you to sustain a healthy diet?”, “How important is it to you to prepare food in a healthy way?”, “How confident are you in your ability to cook food healthily?”, “How prepared are you to cook food in a healthy manner?” The Cronbach’s alpha coefficient for the six items measuring motivation was 0.964. Concurrently, the overall composite reliability (CR) and average variance extracted (AVE) values were 0.964 and 0.818, respectively.

### 2.4. Questionnaire Validity and Reliability Analysis and Model Confirmation

To establish the structural validity of the survey instrument, both exploratory factor analysis (EFA) and CFA were implemented [34,35,36]. Initially, EFA was utilized to identify and extract appropriate measurement items, while unsuitable items were eliminated from further analysis. Subsequently, structural equation modeling was performed using AMOS 23.0 to conduct CFA for validating model fit. Building on this, SEM was developed to examine both the direct and indirect effects among the variables. Additionally, the statistical significance of the mediating effect was confirmed through Bootstrap testing, providing a comprehensive depiction of the mechanisms underlying the relationships among the study variables.

For the exploratory factor analysis, the research employed principal component analysis with factor rotation techniques applied to each domain. Factors with eigenvalues exceeding 1.0 were retained, and a factor loading threshold of 0.60 was established for item classification, with items failing to meet this criterion being removed [37]. The adequacy of sampling was evaluated using Kaiser–Meyer–Olkin (KMO, ≥0.6) measure, Bartlett’s test of sphericity (*p* < 0.05), and examination of “total variance explained” [38]. The CFA utilizing optimal fit SEM was estimated through maximum likelihood procedures, with model fit assessed against several parameters: root mean square error of approximation (RMSEA, ≤0.08) [39], goodness-of-fit index (GFI, ≥0.9), and comparative fit index (CFI, ≥0.9) [40,41].

Internal consistency within each domain of the dietary assessment tool was determined through Cronbach’s alpha reliability testing. Additionally, CR (>0.7) and AVE (>0.5) were calculated to evaluate the measurement model’s reliability and convergent validity. CR serves as an indicator of internal consistency for latent variables, ensuring measurement instrument stability and consistency, while AVE assesses the extent to which latent variables explain observed variable variance, indicating satisfactory convergent validity [42,43].

### 2.5. Statistical Analysis

Continuous data are expressed as mean ± standard deviation (SD), while categorical data are presented as frequencies and percentages. SEM was used to examine the relationships among the three core constructs of the TPB, motivation, and healthy dietary behavior intentions. Statistical analyses were conducted using SPSS software (Statistical Package for the Social Sciences, version 21.0; IBM, Armonk, NY, USA) and AMOS (Analysis of Moment Structures, version 23.0; IBM, Armonk, NY, USA). Statistical significance was defined as *p* < 0.05.

## 3. Results

### 3.1. Demographic Characteristics of Study Participants

A total of 2114 individuals from five Chinese cities participated in and completed the questionnaire. Table 1 presents the demographic characteristics of the population. Among the 2114 subjects, 1202 (56.9%) were female, with a mean age of 33.82 ± 11.68 years and a mean body mass index (BMI) of 22.45 ± 4.94 kg/m^2^ (Table 1).

### 3.2. CFA Fit Indices

The CFA fit indices (Table 2) demonstrated CMIN/DF = 12.728, GFI = 0.841, Tucker–Lewis fit index (TLI) = 0.933, CFI = 0.939, and RMSEA = 0.075. These values indicate that, with the exception of CMIN/DF and GFI values, the model exhibited a “good” fit overall.

### 3.3. Item Selection and Final Scales for TPB and Motivation

#### 3.3.1. Exploratory and Confirmatory Factor Analysis of the TPB and Motivation

Table 3 presents the results of exploratory and confirmatory factor analyses for the TPB constructs, including attitude, subjective norms, PBC, behavioral intention, and motivation. The six items measuring attitude were categorized into a single factor with the following statistical indicators: eigenvalue = 4.814, cumulative variance = 80.237%, KMO = 0.926, Bartlett’s test of sphericity χ^2^ = 12,296.16 (*p* < 0.001), Cronbach’s alpha = 0.950, CR = 0.9508, and AVE = 0.7633. For subjective norms, eight items were classified into one factor with statistical indicators as follows: eigenvalue = 5.937, cumulative variance = 74.207%, KMO = 0.942, Bartlett’s test of sphericity χ^2^ = 15,286.112 (*p* < 0.001), Cronbach’s alpha = 0.950, CR = 0.9504, and AVE = 0.7057. The four items measuring PBC formed a single factor characterized by: eigenvalue = 3.116, cumulative variance = 77.910%, KMO = 0.825, Bartlett’s test of sphericity χ^2^ = 5570.444 (*p* < 0.001), Cronbach’s alpha = 0.905, CR = 0.9063, and AVE = 0.7076. Behavioral intention was represented by four items constituting one factor with: eigenvalue = 3.381, cumulative variance = 84.520%, KMO = 0.866, Bartlett’s test of sphericity χ^2^ = 7587.468 (*p* < 0.001), Cronbach’s alpha = 0.939, CR = 0.9399, and AVE = 0.7964. The six items measuring motivation were grouped into a single factor with statistical indicators: eigenvalue = 5.093, cumulative variance = 84.883%, KMO = 0.907, Bartlett’s test of sphericity χ^2^ = 15,806.100 (*p* < 0.001), Cronbach’s alpha = 0.964, CR = 0.9643, and AVE = 0.8183.

#### 3.3.2. Descriptive Statistics of Constructs of the TPB of Healthy Eating and Healthy Eating Motivation

The average values of attitude questions ranged from 3.10 (“Overall, a healthy diet life is interesting.”) to 4.11 (“Overall, a healthy diet life is good.”) and the total mean value was 4.04 (Table 2). The mean scores of the subjective norm ranged from 3.87 (“Friends and colleagues think I should live a healthy diet”) to 3.98 (“Family members think I should engage in a healthy diet life.”) and the average value was 3.89. The mean scores of the PBC were lowest at “No matter how difficult it is, I will practice a healthy diet life.” (3.73) and highest at “I will try my best to follow a healthy diet life.” (3.96) and the average value was 3.87. The average value of statements of behavioral intention to eat healthily ranged from 3.74 (“I plan to have a healthy diet in the next two weeks.”) to 3.83 (“I want to recommend a healthy diet to friends, family, and colleagues.”) and the total mean was 3.78. The average value of statements of motivation ranged from 6.54 (“How prepared are you for cooking food healthily?”) to 7.51 (“How important is a healthy diet life to you?”) and the total average was 6.90 (Table 3).

#### 3.3.3. Correlations Among Major Variables in the Extended TPB

The correlations among attitude, subjective norms, PBC, motivation, and behavioral intention were as follows: attitude and subjective norms (0.854), attitude and PBC (0.806), attitude and motivation (0.399), attitude and behavioral intention (0.708); subjective norms and PBC (0.862), subjective norms and motivation (0.412), subjective norms and behavioral intention (0.778); PBC and motivation (0.513), PBC and behavioral intention (0.937), and motivation and behavioral intention (0.495). Furthermore, discriminant validity was established through the Fornell–Larcker criterion, confirming that the square root of the AVE for each latent variable exceeded its correlation coefficients with other variables, except for the correlation between subjective norms and PBC (0.862), which was slightly higher than the square root of subjective norms’ AVE (0.8399). These findings support the distinctiveness of each construct, enhance the structural validity of the measurement model, and underscore its applicability and robustness in empirical research (Table 4).

### 3.4. Descriptive Standardized Path Coefficients for the SEM

As shown in Table 5, attitude and subjective norms significantly impacted motivation. The path coefficient for attitude to motivation was 0.14 (standard errors (SE) = 0.043, critical ratio = 3.318, *p* < 0.001), and the path coefficient for subjective norms to motivation was 0.29 (SE = 0.045, critical ratio = 6.574, *p* < 0.001). Additionally, the path coefficient from motivation to behavioral intention was 0.08 (SE = 0.014, critical ratio = 5.625, *p* < 0.001). The path coefficient from PBC to behavioral intention was 0.94 (SE = 0.020, critical ratio = 47.164, *p* < 0.001). Meanwhile, motivation mediates the relationship between attitude and behavioral intention. The indirect effect was found to be standardized indirect effect = 0.011, SE = 0.004, *p* = 0.004, with a 95% CI of 0.004 to 0.021. Similarly, motivation also mediates the relationship between subjective norms and behavioral intention. The indirect effect for this relationship was standardized indirect effect = 0.022, SE = 0.006, *p* = 0.012, with a 95% CI of 0.013 to 0.035 (Table 6).

**Figure 2:**
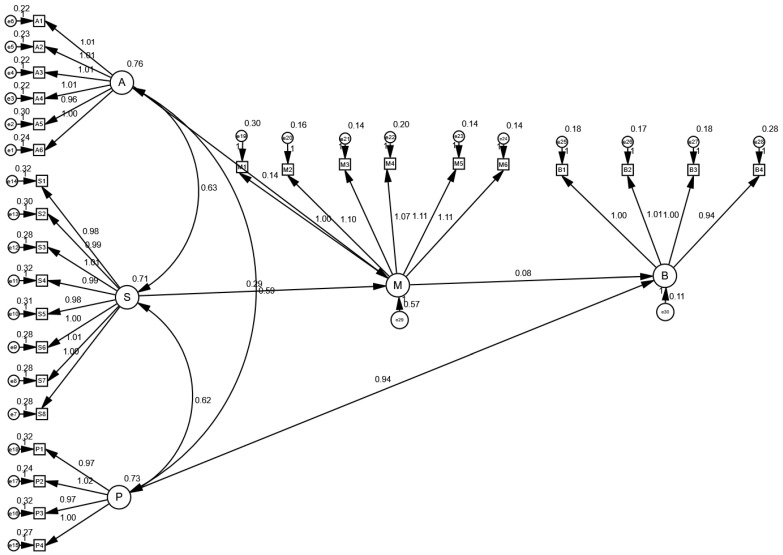
Path diagram.

**Figure 3:**
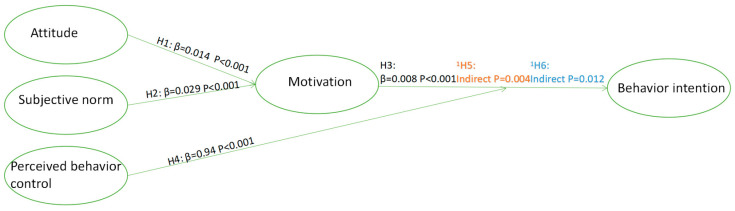
Descriptive standardized path coefficients for the SEM. ^1^ Path analysis diagram. Orange is used to highlight the indirect effect of attitude on behavioral intention through motivation; blue is used to highlight the indirect effect of subjective norms on behavioral intention through motivation.

In this study, to more intuitively illustrate the relationships between variables, we constructed an SEM and generated corresponding path diagrams (Figure 2). These path diagrams demonstrate the direct and indirect relationships between independent and dependent variables, as well as the magnitude and significance levels of each path coefficient (Figure 3). Through these path diagrams, we can clearly observe the differential relationships among variables, providing important visual support for further analysis.

These findings support hypotheses H1–H6, emphasizing the central role of intrinsic motivation in shaping healthy eating intentions among Chinese adults. The results establish a clear pathway relationship: attitudes and subjective norms indirectly influence healthy eating intentions by enhancing individuals’ intrinsic motivation. Notably, motivation plays a significant mediating role in the mechanism by which attitudes and subjective norms affect intentions, further illustrating the critical bridging position of intrinsic motivation between social cognitive factors and behavioral decision making. This finding provides theoretical support for the design of nutritional intervention policies, highlighting the key significance of stimulating individual intrinsic motivation for maintaining healthy behaviors in the long term, particularly against the backdrop of nutritional transition and rapidly changing lifestyles (Table 7).

## 4. Discussion

This study employed an online survey conducted between 2019 and 2023 to examine the relationships among attitudes, subjective norms, PBC, motivation, and healthy eating intentions among adults residing in five Chinese cities: Shanghai, Beijing, Anhui, Henan, and Daqing. SEM revealed significant associations between both attitude and subjective norms with motivation. Furthermore, motivation and PBC were significantly associated with behavioral intention. Specifically, motivation (SE = 0.014, critical ratio = 5.625, *p* < 0.001) and PBC (SE = 0.020, critical ratio = 47.167, *p* = 0.012) both showed strong predictive value. Motivation also mediated the effects of attitude (standardized indirect effect = 0.011, SE = 0.004, *p* = 0.004; 95% CI: 0.004–0.021) and subjective norms (standardized indirect effect = 0.022, SE = 0.006, *p* < 0.001; 95% CI: 0.013–0.035) on behavioral intention. These findings highlight the central role of motivation in the pathway from social–cognitive factors to behavioral intention. Individuals who perceive healthy eating as important and experience social support are more likely to be intrinsically motivated and, subsequently, more likely to form strong intentions to adopt healthy eating behaviors. Thus, motivation acts as a psychological bridge linking external influences and internal decision making.

Our findings are consistent with previous studies conducted in Beijing [24], Daqing [26], and Anhui and Shanghai [25], which identified PBC as the strongest determinant of healthy eating intentions. The current results further confirm that individuals with greater perceived behavioral control—those who anticipate fewer obstacles and perceive more resources—are more likely to form and act upon intentions to eat healthily [44]. Therefore, improving structural support and enhancing individuals’ perceived ease of engaging in healthy eating may increase actual behavior change. A significant positive association was also found between motivation and healthy eating intention, aligning with previous research that identifies motivation as a key determinant of behavior [45,46]. Recent perspectives emphasize that motivation extends beyond pleasure or enjoyment, also encompassing goal-directed processes. When healthy eating is perceived as a means to achieve valued outcomes, such as improved physical health, reduced disease risk, or family well-being, it is more likely to elicit sustained behavioral commitment [17]. In this sense, intrinsic motivation is not merely a subjective preference but an internalized force shaped by personal relevance and perceived health risk, particularly salient in a population where nearly half of adults are overweight or obese [8]. Therefore, enhancing awareness of the long-term benefits of healthy eating may further stimulate goal-aligned motivation and foster durable behavioral change.

Our findings regarding attitude are in line with studies among Dutch families and Australian undergraduates [47,48], suggesting that positive attitudes toward healthy eating enhance motivation and, subsequently, behavioral intention. As attitude reflects personal evaluation of the behavior [23], cultivating positive perceptions of healthy eating should remain a central focus of public health campaigns. In contrast, results concerning subjective norms showed some variability across studies. In our sample, subjective norms significantly influenced motivation and intention via motivational mediation. However, prior research in medium-sized Dutch cities found no significant relationship [47], while studies in Australian samples confirmed such effects [48]. Subjective norms, often shaped by perceived social pressure and the desire to meet others’ expectations [47], may vary in impact depending on sociocultural context. In China, a collectivist society with strong cultural emphasis on social harmony and conformity, individuals may be particularly influenced by normative expectations. As emphasized by President Xi Jinping, “The unity of thought, will, and action is the fundamental reason for China’s continued strength” [49]. This cultural backdrop may help explain the stronger role of subjective norms in our Chinese sample, though direct cross-cultural comparisons must be approached cautiously due to methodological differences.

Consistent findings across several studies have shown that, even after controlling for covariates in the TPB model, PBC continues to exert a significant influence on eating behavioral intention, whereas the effects of attitude and subjective norms are often inconsistent [24,25,26,50,51]. These stable patterns suggest that regional differences may not play a major role in shaping intention. Instead, cultural factors, such as collectivist versus individualist values, may be more relevant in explaining variations in health behavior formation. Future research should, therefore, prioritize cultural dimensions over geographic distinctions when refining TPB-based models. Building on this understanding, the following practical implications are suggested. Since PBC has played a significant role in enhancing the dependent variable, eating behavioral intention, strategies for increasing PBC may be beneficial. Sharing successful cases of individuals who have maintained a healthy diet could trigger dietary intentions. These forms of sharing not only enhance PBC but also have the added advantage of increasing subjective norms, the second most significant predictor in this study. By sharing successful examples, individuals who are exposed to these cases may, in turn, share them with others in their social circles, creating a snowball effect. This ripple strategy can be considered an effective practical approach to promoting a healthy diet across broader populations.

While these practical strategies hold promise, several limitations should be noted. First, motivation could be classified into autonomous and controlled motivation [52]. However, we only included autonomous motivation in our study. Therefore, further studies are needed to explore motivation intensively and how different motivation affects behavioral intention in distinct ways, particularly in cross-cultural settings where motivational patterns may diverge. Second, due to the nature of a cross-sectional study, our study cannot establish causality; future longitudinal studies are needed to confirm causal relationships. Third, within the TPB framework, future studies should employ covariate-adjusted models controlling for potential confounding factors to explore both the mediation effects and potential effect modification of motivation on behavioral intention. Despite these limitations, this study offers several strengths. Unlike prior studies limited to single-city data [24,25,26], this research draws on data from five diverse regions, offering a more comprehensive picture of healthy eating intentions among Chinese adults. Additionally, this study employed SEM, a sophisticated analytical technique that allows for simultaneous testing of multiple pathways, providing a more robust understanding than traditional regression approaches. Importantly, this study is among the first to incorporate motivation as a mediating variable within the TPB framework, elucidating the psychological mechanisms through which attitudes and subjective norms shape behavioral intention.

Future research may explore several directions. First, validation of this extended TPB model across larger and more diverse populations is needed, particularly to examine potential heterogeneity by socioeconomic status, age, or health condition. Second, future studies should consider disaggregating motivation into autonomous, controlled, and motivational subtypes to explore their differential effects on intention and behavior. Third, longitudinal designs could track how changes in motivation relate to the maintenance of healthy eating over time, providing insight into sustained behavior change. Such findings would inform the development of tailored, motivation-centered nutrition interventions that leverage digital tools, social incentives, and supportive environments. Bridging theoretical insight and practical application will be essential to improving population-level dietary behaviors and advancing chronic disease prevention in China.

## 5. Conclusions

This study investigated the role of motivation in shaping healthy eating intentions within an extended TPB framework. Amid China’s rapid economic growth and urbanization, the rising prevalence of obesity has emerged as a critical public health concern, increasing the risk of chronic diseases such as hypertension, type 2 diabetes, and cardiovascular conditions and negatively impacting both physical and mental health. Promoting healthy dietary behaviors is, thus, essential to mitigating these risks. An online survey conducted between 2019 and 2023 collected data from 2114 adults across several regions in China. SEM showed that attitudes and subjective norms were significantly associated with motivation, while motivation and PBC strongly predicted healthy eating intentions. Motivation also mediated the effects of attitudes and subjective norms on intention, highlighting its key intermediary role. The SEM results revealed that attitudes and subjective norms were significantly associated with motivation, while both motivation and PBC strongly predicted healthy eating intentions. Moreover, motivation mediated the effects of attitudes and subjective norms on behavioral intention, as confirmed by statistically significant indirect effects. These findings suggest that attitudes and social expectations alone are insufficient to explain intention formation; motivation is a crucial intermediary that bridges external influences and internal commitment. Importantly, intrinsic motivation appears to stem not only from identification with healthy eating behaviors themselves but also from their perceived utility in achieving personally meaningful goals. When individuals recognize that healthy eating contributes to improved health or quality of life, they are more likely to sustain behavioral intention and engagement. This insight broadens the explanatory scope of TPB and offers theoretical grounding for the design of more targeted and motivation-enhancing interventions. In the context of complex food environments and accelerated lifestyles, fostering motivation for healthy eating should be a central focus of future intervention strategies. Policymakers are encouraged to apply behavioral science tools, such as personalized health feedback, social incentive structures, and digital health platforms, to promote the adoption and maintenance of healthy eating practices. Tailored strategies that address the diverse needs of different socioeconomic groups are also essential to reducing health disparities. By combining motivation-centered interventions with long-term public health initiatives, it is possible to create a supportive social environment for healthy eating, reduce the burden of obesity and chronic diseases, and improve overall population health.

## Figures and Tables

**Table 1:** Demographic characteristics of study participants, China, 2019–2023 (*n* = 2114).

Variables	Values
	n (%)
Female	1202 (56.9)
Age (years)	
18–29	954 (45.1)
30–39	486 (23.0)
40–49	401 (19.0)
50–64	273 (12.9)
Mean ± SD	33.82 ± 11.68
Body mass index (kg/m^2^)	
<18.5	296 (14.0)
18.5–22.99	1033 (48.9)
23–24.99	374 (17.7)
≥25	411 (19.4)
Mean ± SD	22.45 ± 4.94

**Table 2:** Confirmatory factor analysis fit indices.

Fitting Index	Value	Ideal Range	Description
CMIN (Chi-square)	4181.503	--	smaller is better
DF (Degrees of Freedom)	340	--	
CMIN/DF	12.299	<5	
GFI (Goodness-of-Fit Index)	0.850	≥0.90	close to 1 indicates good fit.
TLI (Tucker–Lewis Fit Index)	0.936	≥0.90	
CFI (Comparative Fit Index)	0.942	≥0.90	
RMSEA (Root Mean Square Error)	0.073	≤0.08	lower values indicate better fit.

**Table 3:** Exploratory and confirmatory factor analysis of the TPB and motivation.

Exploratory Factor Analysis				
Attitude (A)	Item	Factor 1	Factor loading	Mean ± SD
	In general, living a healthy diet is advantageous.(A1)	0.906	0.886	4.11 ± 0.86
	On the whole, maintaining a healthy diet is valuable.(A2)	0.900	0.880	4.08 ± 0.86
	Overall, following a healthy diet is positive.(A3)	0.905	0.885	4.07 ± 0.87
	In general, adopting a healthy diet is satisfying.(A4)	0.904	0.883	4.03 ± 0.90
	Overall, leading a healthy diet is engaging.(A5)	0.864	0.834	3.98 ± 0.90
	On the whole, embracing a healthy diet is something to look forward to.(A6)	0.896	0.873	3.10 ± 0.89
	Eigen value = 4.814, Cumulative variance (%) = 80.237%			
	KMO = 0.926, Bartlett’s test of sphericity χ^2^ = 12,296.16 (*p* < 0.001)			
	Cronbach alpha = 0.950			
	CR = 0.9508, AVE = 0.7633			
Subjective norm (S)	Item	Factor 1	Factor loading	Mean ± SD
	Family members believe that I should engage in a healthy dietary lifestyle.(S1)	0.840	0.826	3.98 ± 0.89
	Friends believe that I should engage in a healthy dietary lifestyle.(S2)	0.852	0.838	3.92 ± 0.92
	Experts (such as doctors, nutritionists, etc.) believe that I should engage in a healthy dietary lifestyle.(S3)	0.868	0.850	3.87 ± 0.92
	The government believes that I should engage in a healthy dietary lifestyle.(S4)	0.853	0.827	3.92 ± 0.92
	Television programs (including those watched online) believe that I should engage in a healthy dietary lifestyle.(S5)	0.862	0.834	3.87 ± 0.96
	Newspapers and magazines (including those read online) believe that I should engage in a healthy dietary lifestyle.(S6)	0.873	0.847	3.88 ± 0.95
	Online information (such as Weibo, TikTok, etc.) believes that I should engage in a healthy dietary lifestyle.(S7)	0.876	0.852	3.88 ± 0.94
	Family members believe that I should engage in a healthy dietary lifestyle.(S8)	0.867	0.846	3.87 ± 0.93
	Eigen value = 5.937, Cumulative variance (%) = 74.207%			
	KMO = 0.942, Bartlett’s test of sphericity χ^2^ = 15,286.112 (*p* < 0.001)			
	Cronbach alpha = 0.950			
	CR = 0.9504, AVE = 0.7057			
Perceived behavioral control (P)	Item	Factor 1	Factor loading	Mean ± SD
	I will make an effort to follow a healthy dietary lifestyle.(P1)	0.860	0.824	3.96 ± 0.86
	I am actively practicing a healthy dietary lifestyle.(P2)	0.907	0.866	3.86 ± 0.93
	I have sufficient time to practice a healthy dietary lifestyle.(P3)	0.875	0.822	3.75 ± 0.97
	Regardless of how difficult it may be, I will adhere to a healthy dietary lifestyle.(P4)	0.889	0.852	3.73 ± 0.98
	Eigen value = 3.116, Cumulative variance (%) = 77.910%			
	KMO = 0.825, Bartlett’s test of sphericity χ^2^ = 5570.444 (*p* < 0.001)			
	Cronbach alpha = 0.905			
	CR = 0.9063, AVE = 0.7076			
Behavioral intention (B)	Item	Factor 1	Factor loading	Mean ± SD
	I intend to adopt a healthy diet within the next two weeks.(B1)	0.926	0.904	3.78 ± 0.94
	I want to adopt a healthy diet within the next two weeks.(B2)	0.933	0.913	3.77 ± 0.96
	I plan to adopt a healthy diet within the next two weeks.(B3))	0.928	0.905	3.74 ± 0.99
	I wish to recommend a healthy diet to friends, family, and colleagues.(B4)	0.889	0.846	3.83 ± 0.95
	Eigen value = 3.381, Cumulative variance (%) = 84.520%			
	KMO = 0.866, Bartlett’s test of sphericity χ^2^ = 7587.468 (*p* < 0.001)			
	Cronbach alpha = 0.939			
	CR = 0.9399, AVE = 0.7964			
Motivation (M)	Item	Factor 1	Factor loading	Mean ± SD
	How important is maintaining a healthy diet to you?(M1)	0.877	0.834	7.51 ± 2.66
	How confident are you in your ability to maintain a healthy diet?(M2)	0.931	0.915	6.78 ± 2.70
	How ready are you to sustain a healthy diet?(M3)	0.934	0.926	6.56 ± 2.81
	How important is it to you to prepare food in a healthy way?(M4)	0.923	0.896	7.23 ± 2.65
	How confident are you in your ability to cook food healthily?(M5)	0.932	0.926	6.75 ± 2.75
	How prepared are you to cook food in a healthy manner?(M6)	0.930	0.927	6.54 ± 2.84
	Eigen value = 5.093, Cumulative variance (%) = 84.883%			
	KMO = 0.907, Bartlett’s test of sphericity χ^2^ = 15,806.100 (*p* < 0.001)			
	Cronbach alpha = 0.964			
	CR = 0.9643, AVE = 0.8183			

**Table 4:** The square root of the average variance extracted (AVE) and correlation coefficients.

	Attitude	Subjective Norm	Perceived Behavioral Control	Motivation	Behavioral Intention
Attitude	0.8737 ^1^				
Subjective norm	0.854	0.8399 ^1^			
Perceived behavioral control	0.806	0.862	0.8412 ^1^		
Motivation	0.399	0.412	0.513	0.9046 ^1^	
Behavioral intention	0.708	0.778	0.937	0.495	0.8924 ^1^

^1^ The figures displayed on the diagonal of the table represent the square roots of the AVE for each construct.

**Table 5:** Descriptive standardized path coefficients for the structural equation model.

Path			Estimate	SE	Critical Ratio.	*p*	β	Support Label
Attitude	--->	Motivation	0.143	0.043	3.318	***	0.14	H1
Subjective norm	--->	Motivation	0.294	0.045	6.574	***	0.29	H2
Motivation	--->	Behavioral intention	0.079	0.014	5.625	***	0.08	H3
Perceived behavioral control	--->	Behavioral intention	0.943	0.020	47.164	***	0.94	H4

*** *p* ≤ 0.001.

**Table 6:** Mediating effects.

Path					Estimate	Standardized Indirect Effect	SE	*p*	95% CI		Support Label
									Lower Bounds	Upper Bounds	
Attitude	--->	Motivation	--->	Behavioral intention	Indirect	0.011	0.004	0.002	0.004	0.021	H5
Subjective norm	--->	Motivation	--->	Behavioral intention	Indirect	0.022	0.006	0.012	0.013	0.035	H6

**Table 7:** Hypothesis verification results.

Hypothesis	Code	Proven Situation
Attitudes have a significant effect on motivation.	H1	Proven
Subjective norms have a significant effect on motivation.	H2	Proven
Motivation has a significant effect on healthy eating intentions.	H3	Proven
Perceived behavioral control has a significant effect on healthy eating intentions.	H4	Proven
Motivation mediates the relationship between attitudes and healthy eating intentions.	H5	Proven
Motivation mediates the relationship between subjective norms and healthy eating intentions.	H6	Proven

## Data Availability

The data are not publicly available due to privacy, access to data can be requested from the corresponding author.
